# Supplementary material for: No evidence for basigin/CD147 as a direct SARS-CoV-2 spike binding receptor
Source: Sci Rep. 2021 Jan 11;11:413. doi: 10.1038/s41598-020-80464-1 (PMC7801465; doi:10.1038/s41598-020-80464-1)
Supplement: Supplementary file 1 — Supplementary Figures. [file 41598_2020_80464_MOESM1_ESM.pdf]

Supplementary information for

"No evidence for basigin/CD147 as a direct SARS-CoV-2 spike binding receptor"

Jarrod Shilts, Thomas W. M. Crozier, Edward J. D. Greenwood,
Paul J. Lehner, and Gavin J. Wright

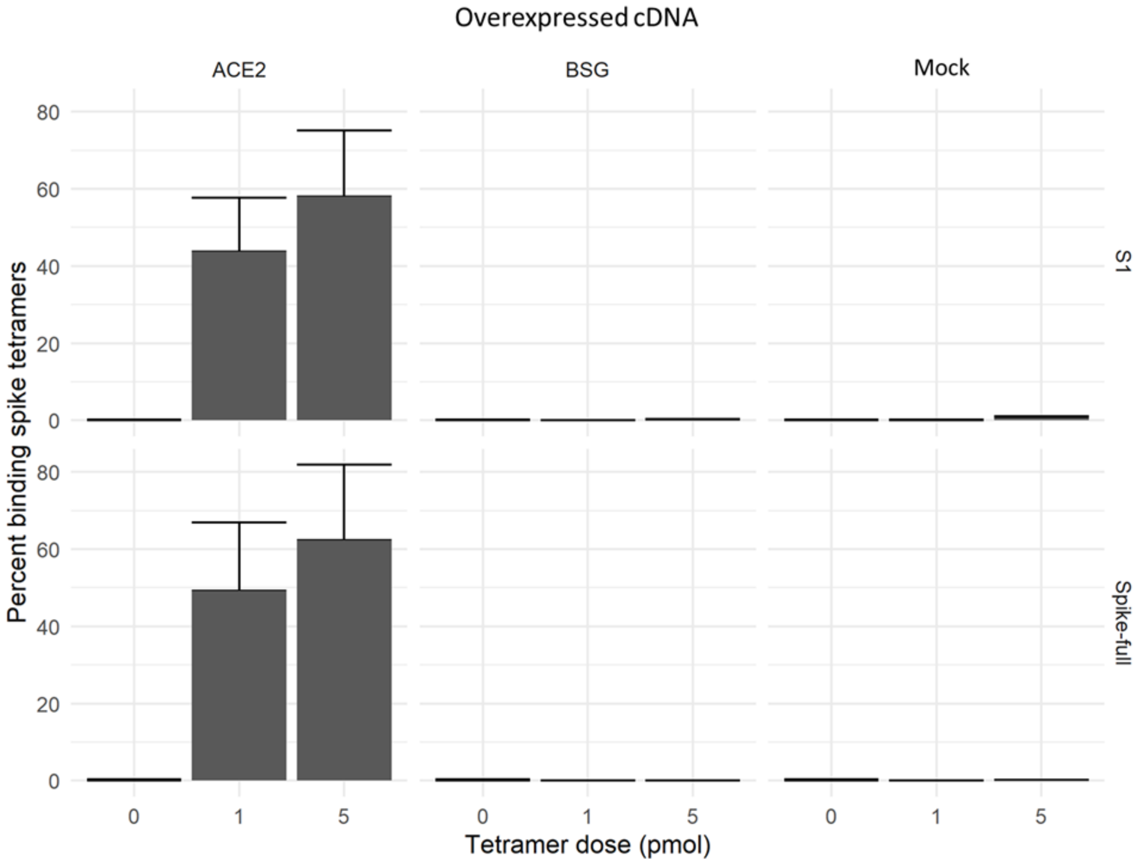

Figure S1. Quantified percentages of cells positively-stained by SARS-CoV-2 spike-protein tetramers. HEK293 cells were transfected with cDNA expression constructs encoding either ACE2 or BSG and the percentages of cells that were stained with a highly-avid fluorescently labeled SARS-CoV-2 spike S1 domain quantified by flow cytometry and compared to a mock transfected control. Bars show means + standard deviation; *n* = 3.

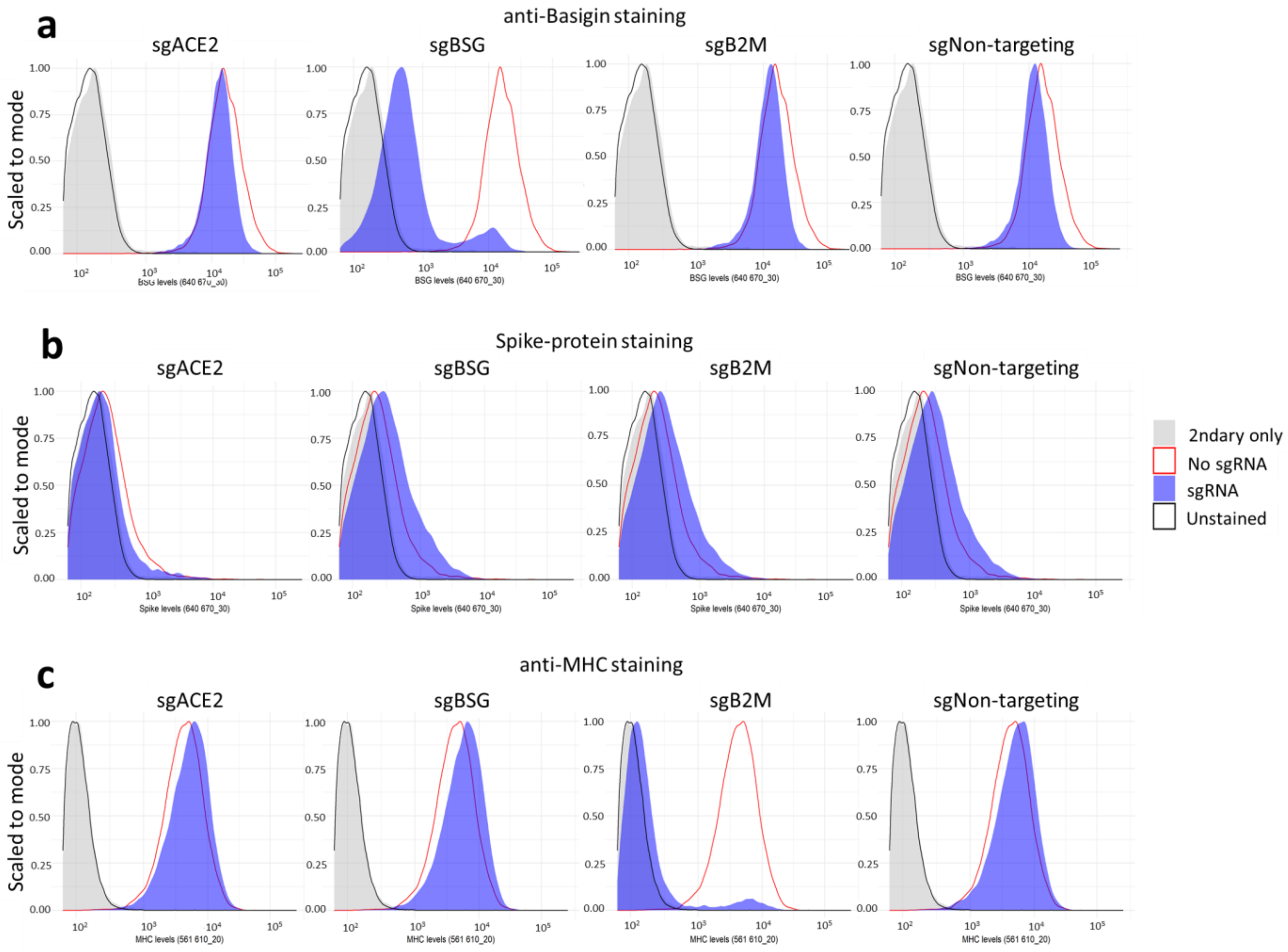

Figure S2. CRISPR-Cas9 editing in CaLu-3 cells leads to specific reductions in the targeted cell-surface receptors. Unedited Cas9-expressing control cells are shown as red distributions, while cells transduced with guide RNAs against the indicated gene are shown in blue (**a** - basigin antibody, **b** - spike staining, **c** - MHC class I antibody). Negative controls are shown in grey and black for secondary-antibody only staining and unstained cells respectively.

Supplementary uncropped gel images

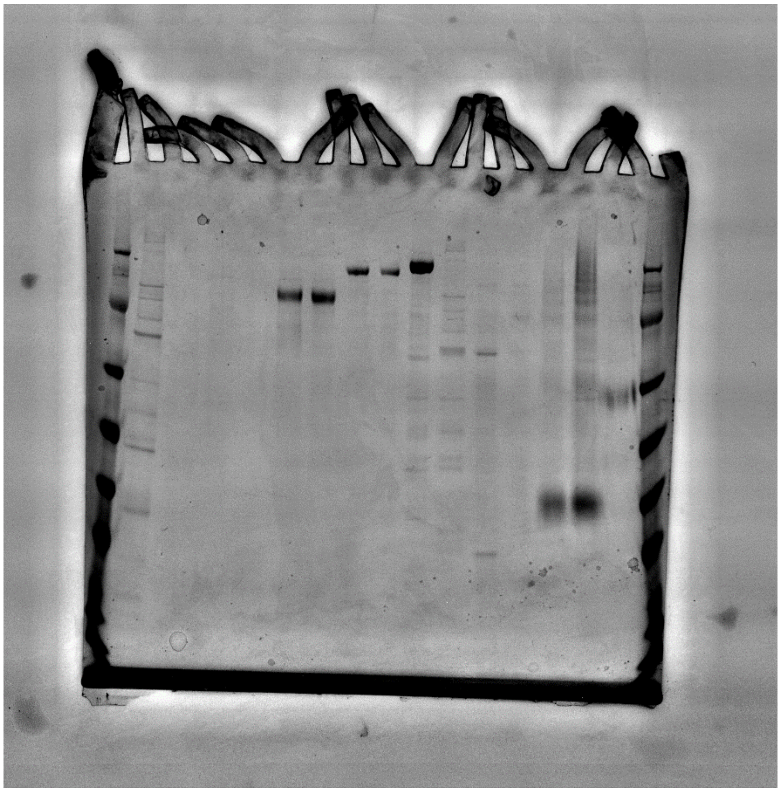

Full Coomassie gel corresponding to **Figure 1a**. Bands not included in the main-text figure correspond to unrelated proteins from separate and unrelated projects.

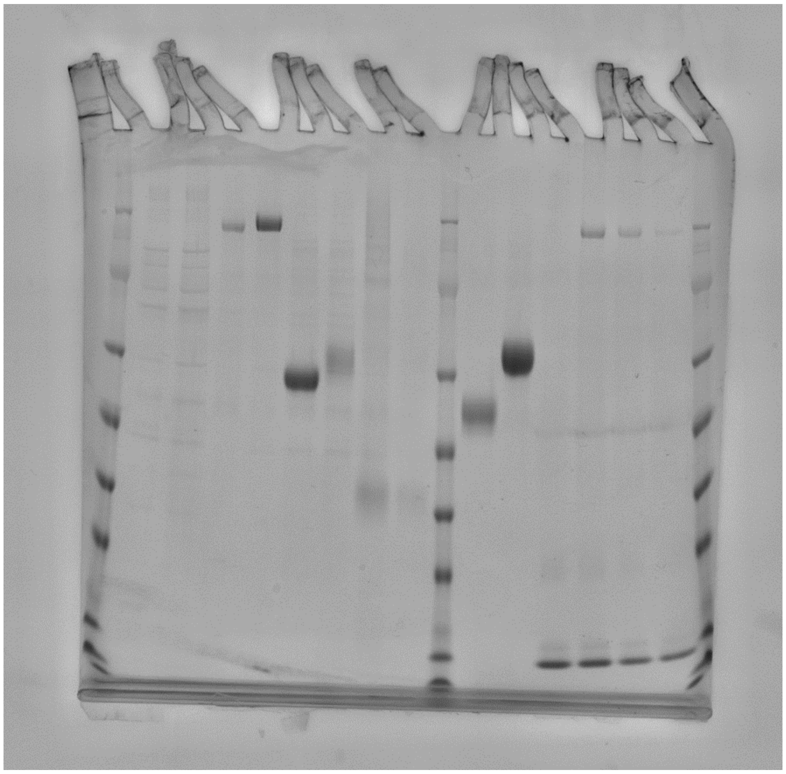

Full Coomassie gel corresponding to **Figure 2a**. Bands not included in the main-text figure correspond to unrelated proteins from separate and unrelated projects.
